# Evaluation of Cyclotron Solid Target Produced Gallium-68 Chloride for the Labeling of [^68^Ga]Ga-PSMA-11 and [^68^Ga]Ga-DOTATOC

**DOI:** 10.3390/molecules30173458

**Published:** 2025-08-22

**Authors:** Michał Jagodziński, Jakub Boratyński, Paulina Hamankiewicz, Łukasz Cheda, Witold Uhrynowski, Agnieszka Girstun, Joanna Trzcińska-Danielewicz, Zbigniew Rogulski, Marek Pilch-Kowalczyk

**Affiliations:** 1 VOXEL S.A, Radiopharmaceutical Production Center, ul. Wrocławska 1-3, 30-006 Kraków, Poland; j.boratynski@voxel.pl (J.B.); p.hamankiewicz@voxel.pl (P.H.); mpk@voxel.pl (M.P.-K.); 2 University of Warsaw, Faculty of Chemistry, Pasteura 1, 02-093 Warsaw, Poland; lcheda@chem.uw.edu.pl (Ł.C.); rogul@chem.uw.edu.pl (Z.R.); 3 University of Warsaw, Faculty of Biology, Miecznikowa 1, 02-096 Warsaw, Poland; a.girstun@uw.edu.pl (A.G.); j.trzcinska-da@uw.edu.pl (J.T.-D.)

**Keywords:** cyclotron, gallium-68, [^68^Ga]Ga-PSMA-11, [^68^Ga]Ga-DOTATOC

## Abstract

Gallium-68 is a widely used positron-emitting radionuclide in nuclear medicine, traditionally obtained from ^68^Ge/^68^Ga generators. However, increasing clinical demand has driven interest in alternative production methods, such as medical cyclotrons equipped with solid targets. This study evaluates the functional equivalence of gallium-68 chloride obtained from cyclotron solid target and formulated to be equivalent to the eluate from a germanium-gallium generator, aiming to determine whether this production method can serve as a reliable alternative for PET radiopharmaceutical applications. Preparations of [^68^Ga]Ga-PSMA-11 and [^68^Ga]Ga-DOTATOC, labeled with cyclotron-derived gallium-68 chloride, were subjected to quality control analysis using radio thin layer chromatography and radio high performance liquid chromatography. Subsequently, biodistribution studies were performed in mouse oncological models of expression of PSMA antigen and SSTR receptor to compare uptake of preparations produced with generator and cyclotron-derived isotopes. All tested formulations met the required radiochemical purity specifications. Moreover, tumor accumulation of the radiolabeled compounds was comparable regardless of the isotope source. The results support the conclusion that gallium-68 produced via cyclotron is functionally equivalent to that obtained from a generator, demonstrating its potential for interchangeable use in clinical and research radiopharmaceutical applications.

## 1. Introduction

In recent years, the number of PET examinations using radiopharmaceuticals labeled with gallium-68 isotope has been steadily increasing [1]. Currently, the primary clinical indications for such radiopharmaceuticals include the diagnosis of prostate cancer and neuroendocrine tumors, using compounds targeting the prostate-specific membrane antigen (PSMA) [2] and somatostatin receptors (SSTR) [3], respectively. The increased demand for gallium-68 isotope is also associated with subsequent registrations of medicinal products of kits for radiopharmaceutical preparations: NETSPOT^TM^ (Advanced Accelerator Applications), approved in June 2016 in the United States [4], SomaKit TOC^®^ (Advanced Accelerator Applications), approved in December 2016 in Europe [5,6], Illuccix^®^ (TelixPharma), approved in December 2021 in the United States [7], and Locametz^®^ (Advanced Accelerator Applications), approved in April 2022 in the United States [8] and in December 2022 in Europe [9]. ^68^Ge/^68^Ga generators are currently the main source of the gallium-68 isotope, but they have some disadvantages, such as high purchase cost, expiry date below 1 year, low maximum and gradually deceasing activity of the obtained isotope, and inelastic pattern of successive elutions [10]. Significant efforts are underway to introduce cyclotron-produced gallium-68 into clinical practice via nuclear reaction ^68^Zn(p,n)^68^Ga. This can be achieved by irradiating either a liquid target, i.e., a solution with the zinc-68 isotope, or a solid target, where metallic zinc-68 is deposited on a solid backing [11]. Compared to a generator, the liquid target method yields gallium-68 isotope with a 2–3 times higher activity [12], whereas the solid target can provide 10–15 times higher activity [13]. However, the latter method presents additional challenges, including extended target preparation time, activation of the target backing, and the need for an additional dissolution step [14]. In the last decade, two radiopharmaceutical precursors, GalliUC (University of Coimbra), produced using liquid target technology [15], and a more recent V-Ga68 (VOXEL), described in this work, derived from a solid target [16], have been registered.

This study aimed to develop a radiopharmaceutical precursor suitable for centralized production and distribution, thereby facilitating broader access to gallium-68-based diagnostics in settings without on-site cyclotron infrastructure. A further objective was to demonstrate the functional equivalence of gallium-68 chloride obtained from a generator and from a cyclotron equipped with a solid target. Establishing such equivalence is crucial for the direct use of the isotope with commercially available radiolabeling kits and facilitates the application of existing, optimized procedures without the need for additional modifications.

## 2. Results

This study describes gallium-68 chloride obtained via a cyclotron solid target, formulated to be equivalent to the eluate from a germanium-gallium generator. The developed process enables the production of up to 40 GBq of the final product. Each production batch yielded multiple vials containing 5 mL of gallium-68 chloride, as well as three vials intended for quality control in compliance with the European Pharmacopoeia, monograph 3109 for gallium-68 of cyclotron origin [17].

Radionuclide purity and identity analyses were performed as a part of the tests for gallium-68 chloride and were not performed again for labeled compounds. For all samples, they were less than 2% of the total radioactivity due to gallium 67 and gallium-66 and less than 0.1% for other impurities at the end of the 5-h shelf life of gallium-68 chloride. Additionally, the content of iron and zinc remained below the specified limit of 10 µg/GBq throughout the product’s expiry period, which was in line with the results presented in the above-mentioned monograph. As the product was formulated with a 0.1 M hydrochloric acid solution, the resulting pH value was 1.00 ± 0.05, the same as for the Galliapharm generator. Quality control results of representative batch of gallium-68 chloride produced by VOXEL Radiopharmaceutical Production Center were presented in Table S1 in the Supplementary Materials.

This approach enabled the production of multiple vials of ready-to-use gallium-68 chloride from a single batch, facilitating distribution to multiple end users, while the quality control testing and batch release of the precursor were ongoing. Nuclear medicine departments could then use cold kits or their own established procedures to prepare various radiolabeled compounds for PET/CT imaging. As part of this research, solid target cyclotron-produced gallium-68 chloride was successfully transported over a distance of 300 km, followed by radiolabeling, which would be impossible with isotope activity obtained from a generator or cyclotron liquid target. A similar centralized production and distribution model was previously described by Kumar et al. [18], wherein ready-to-use, generator-derived gallium-68 radiopharmaceuticals were supplied within a 50 km radius.

In our study, two radiopharmaceutical preparations, [^68^Ga]Ga-PSMA-11 and [^68^Ga]Ga-DOTATOC, were evaluated. In the first stage, physicochemical testing of the above-mentioned preparations was performed. Each was synthesized using three different precursors: two in the form of lyophilized peptides and one as a ready-made cold kit for radiolabeling. For each precursor, three independent labeling runs using solid target cyclotron-produced gallium-68 chloride were conducted. Radiolabeling procedures for the individual preparations were selected to align with protocols previously optimized for the isotope obtained from a germanium-68/gallium-68 generator [19,20]. The PSMA-11 precursor contains the HBED chelator, which enables efficient gallium-68 labeling without requiring additional purification [21]. To prevent potential radiolysis of the preparations, ascorbic acid was added to the product as a radiolytic inhibitor. Quality control results for [^68^Ga]Ga-PSMA-11 preparations are summarized in Table 1.

In all the runs, a clear, colorless solution was obtained. The pH value was approximately 4.0, with slightly higher values observed for preparations using the cold kit. Radiochemical purity analysis via TLC and HPLC revealed minimal, negligible differences between preparations. In all samples, the radiochemical purity was higher than the 97% limit required by the pharmacopoeia [22]. The labeling of [^68^Ga]Ga-PSMA-11 was performed using activities that yielded final product activities in the range of 1090–3095 MBq, with most values exceeding 2000 MBq.

Quality control results for [^68^Ga]Ga-DOTATOC preparations are summarized in Table 2. Again, a clear, colorless solution was obtained in all the runs. The pH for manually prepared formulations was above 4.00, whereas the pH for cold-kit preparations was 3.28 ± 0.02, consistent with the kit specifications (range: 3.20–3.80). Radiochemical analyses via TLC and HPLC results were above the 97% limit required by the pharmacopoeia [23]. TLC results showed slightly lower radiochemical purity compared to HPLC. This discrepancy may be due to colloidal gallium-68 being retained on the HPLC column, preventing its detection by the radiodetector [24,25]. The obtained yields were 60.81 ± 1.96% for the first precursor and 54.15 ± 0.40% for the second, which were comparable to the results achieved under analogous labeling conditions using generator-derived gallium-68 (55.1 ± 7.2%) [19].

**Figure 1:**
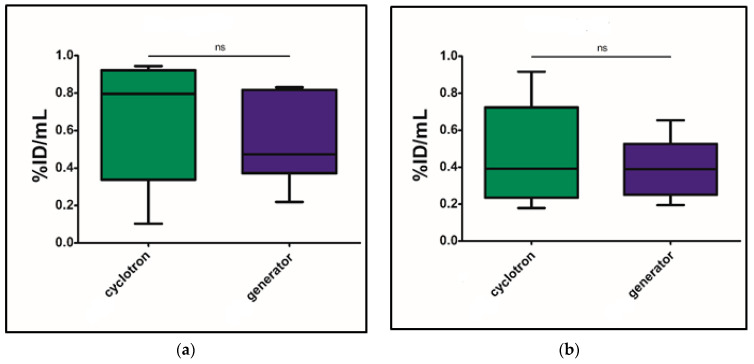
Comparison of accumulation of the tested preparations in tumor tissue: (**a**) comparison of accumulation of [^68^Ga]Ga-PSMA-11 of different origins in the mouse model of expression of PSMA antigen; (**b**) comparison of accumulation of [^68^Ga]Ga-DOTATOC of different origins in the mouse model of expression of SSTR receptors.

The differences in the accumulation of gallium-68 isotope-labeled preparations were analyzed based on the %ID/mL values obtained from PET/CT images 60 min post-injection. The statistical significance of differences between preparations labeled with cyclotron- and generator-derived gallium-68 isotope was evaluated using Student’s t-test for independent samples. No statistically significant differences were observed (*p* > 0.05), as illustrated in Figure 1a for [^68^Ga]Ga-PSMA-11 and Figure 1b for [^68^Ga]Ga-DOTATOC.

**Figure 2:**
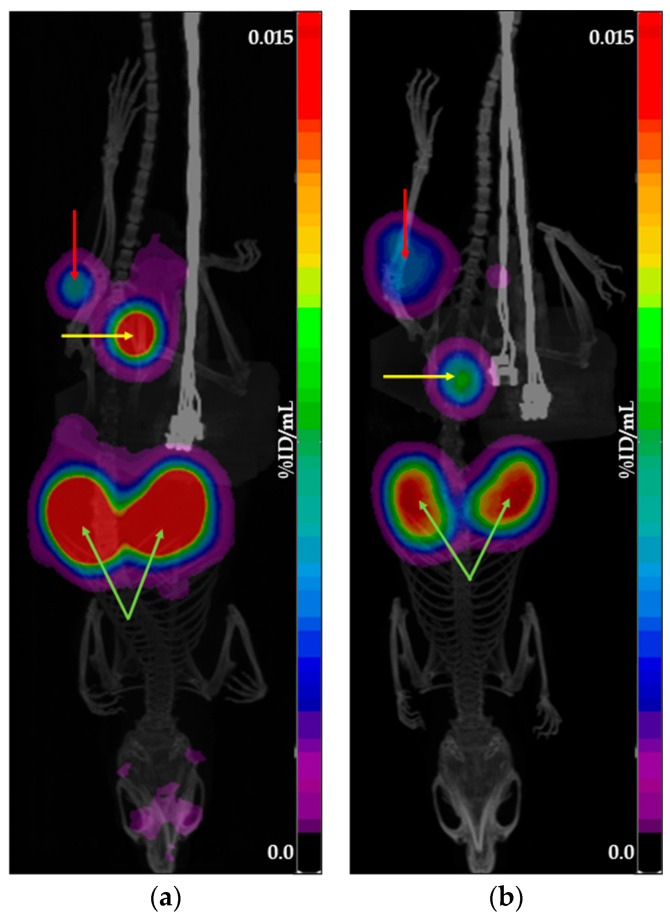
Example of PET/CT scans obtained using [^68^Ga]Ga-PSMA-11: (**a**) produced with cyclotron solid target isotope; (**b**) produced with generator-origin isotope. The images show the main places of tracer accumulation: the red arrow indicates the tumor, the yellow arrow indicates the bladder, and the green arrow indicates the kidneys.

**Figure 3:**
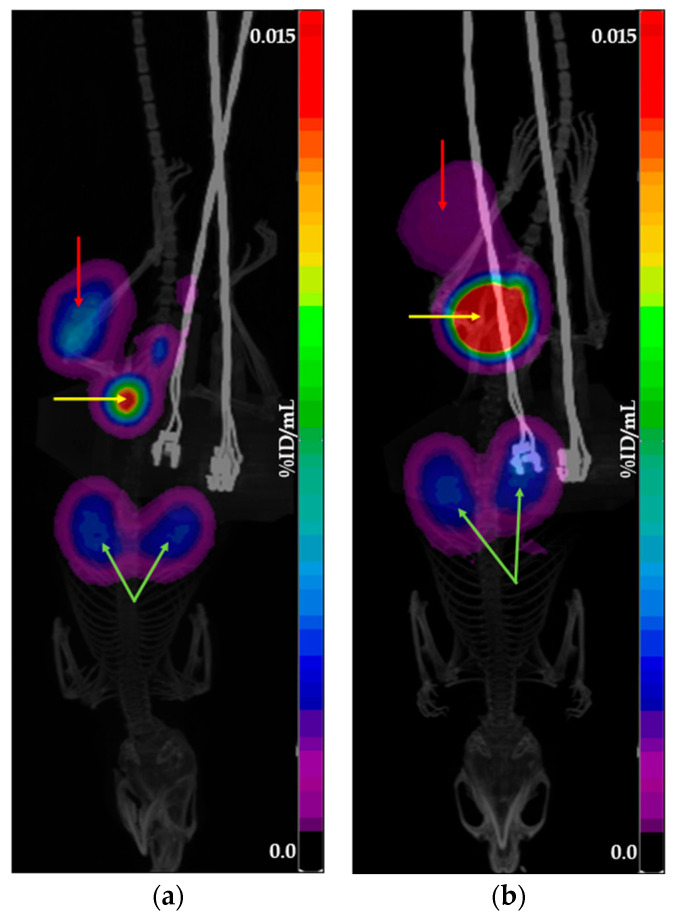
Example of PET/CT scans obtained using [^68^Ga]Ga-DOTATOC: (**a**) produced with cyclotron solid target isotope; (**b**) produced with generator-origin isotope. The images show the main places of tracer accumulation: the red arrow indicates the tumor, the yellow arrow indicates the bladder, and the green arrow indicates the kidneys.

To achieve an activity concentration suitable for in vivo administration, all tested preparations were concentrated. Since the concentration process was identical for both generator- and cyclotron-derived preparations, it is unlikely to have affected the comparative analysis of their tumor accumulation profiles. Figure 2 and Figure 3 show examples of PET/CT scans obtained using [^68^Ga]Ga-PSMA-11 and [^68^Ga]Ga-DOTATOC produced with cyclotron solid target isotope and generator-origin isotopes.

## 3. Discussion

The demand for the gallium-68 isotope has been steadily increasing in recent years. In response to this growing need last year, the GalliaPharm generator (Eckert & Ziegler) was registered in Europe with a maximum activity of 3.70 GBq based on the parent isotope germanium-68 [26]. According to the manufacturer, the elution yield of the generator is not less than 60%, which corresponds to the activity of 2.22 GBq of gallium-68 isotope [26]. Over time, this amount decreases proportionally to the parent isotope half-life and, after 9 months, it is lowered by half. An alternative source of the gallium-68 isotope is medical cyclotron, where it can be obtained in two ways: with the use of a liquid or solid target approach. Unlike generator-derived gallium-68, cyclotron production enables consistent activity of the isotope, ensuring an adequate supply to meet the needs of a maximum number of patients. This approach may be particularly advantageous for nuclear medicine facilities that use gallium-68 isotope in smaller quantities or less frequently. On-demand isotope delivery could help optimize resource utilization and reduce the overall costs.

Numerous reports in the literature describe the cyclotron-based production of gallium-68, focusing primarily on maximizing activity yield and minimizing zinc impurity levels. This suggests that the implementation of cyclotron-produced gallium-68 in clinical practice is still evolving, remains far from standardized, and is currently under active investigation. Most medical cyclotron facilities produce ^68^Ga radiopharmaceuticals exclusively for in-house use [1]. Furthermore, specific guidelines have been established for the production and quality control of gallium-68 in chloride form intended for radiopharmaceutical applications [11,27]. Several studies have reported the successful use of gallium-68 chloride obtained from cyclotron solid targets for radiolabeling various peptides. These studies have explored different precursor and radiostabilizer concentrations, as well as a broad range of starting gallium-68 activities. In each case, the resulting radiopharmaceutical products met the established specification limits, demonstrating the robustness and reliability of cyclotron-produced gallium-68 for clinical applications. For example, Tieu et al. [28] reported the production of 1.56 GBq of [^68^Ga]Ga-DOTATATE through the labeling of 80 μg of precursor in acetate buffer. In Lin and co-workers’ [29] study, 100 μg of PSMA-11 precursor and 200 mg of L-ascorbic acid in acetate buffer were used, yielding 14.06–32.08 GBq of [^68^Ga]Ga-PSMA-11. Thisgaard et al. [30] mixed 50 mg of sodium ascorbate, 100 nmol PSMA-11, and sodium acetate buffer, obtaining, after purification with the use of C18 light column, up to 72.2 GBq of [^68^Ga]Ga-PSMA-11. Svedjehed et al. [31], in a reaction of 8 μg PSMA-11 precursor, 5 mg ascorbic acid, and 0.9 M NaOAc buffer, received up to 42 GBq [^68^Ga]Ga-PSMA-11, and in the single test of the Telix PSMA-11 kit, they added an additional 18 mg of ascorbic acid to the buffer, which allowed to receive 199 GBq of [^68^Ga]Ga-PSMA-11. Jussing et al. [32] performed radiolabeling of [^68^Ga]Ga-DOTATOC and [^68^Ga]Ga-FAPI-46 with the use of solid target gallium-68 chloride. To the reactor containing 50 μg of FAPI-46 or 40 μg DOTATOC precursor and sodium acetate buffer with ethanol, they eluted gallium-68 chloride in sodium chloride/hydrochloric acid solution (final volume 3.3 mL, pH 3.5). After purification on the C18 cartridge, they received 5.58 ± 0.35 GBq for [^68^Ga]Ga-FAPI-46 and 6.1 ± 1.3 GBq for [^68^Ga]Ga-DOTATOC. In a recent study, Wang et al. reported, for the first time, the compatibility of gallium-68 chloride produced using a liquid target in a medical cyclotron with commercially available radiopharmaceutical labeling kits, NETSPOT and Illuccix. However, their approach required a purification process involving five purification cartridges [33]. Additionally, the authors suggested the possibility of diluting their product in a manner analogous to the dilution of sodium pertechnetate-99m solution used in SPECT diagnostics.

In recent years, several reports have been published on the clinical use of radiopharmaceuticals labeled with gallium-68 produced in a cyclotron. In the clinical study by Martinez et al. [34], it was reported that [^68^Ga]Ga-PSMA-11 produced using a cyclotron solid target and that obtained from a generator resulted in equivalent scans for the detection of PSMA-positive lesions. However, the methodology for obtaining the preparations was not provided by the authors. In studies by Tremblay et al. [35] and Chattopadhyay et al. [36], the authors described the production of [^68^Ga]Ga-DOTATATE and [^68^Ga]Ga-PSMA-11 in a continuous process using the same synthesizer in which the cyclotron solid target gallium-68 chloride solution was obtained. In the study by Tremblay et al., 20.7 ± 1.3 GBq of [^68^Ga]Ga-DOTATATE was produced, while Chattopadhyay et al. reported the production of 12.0–12.5 GBq of [^68^Ga]Ga-PSMA-11 and 5.9–6.2 GBq of [^68^Ga]Ga-DOTATATE. Rodnick et al. [37] described a method for producing [^68^Ga]Ga-PSMA-11 using a liquid target, implemented at two clinical sites. Activities ranging from 1.47 to 1.89 GBq were obtained from a single cyclotron target, and up to 3.63 GBq when using dual target. In all cases, high radiochemical purity of the final products was achieved; however, the described procedure differed from the one used for generator-derived gallium-68.

As demonstrated by the examples discussed above, the reported radiolabeling procedures are often specifically tailored to cyclotron-derived gallium-68 and are not compatible with existing kit-based approaches optimized for generator-produced gallium-68. This highlights a gap in the field. To address this issue, we developed a production process and a radiopharmaceutical precursor that can be centrally manufactured and distributed, thereby facilitating broader access to gallium-68-based diagnostics without the need for costly infrastructure. In contrast to previous reports, the present study introduces a ready-to-use formulation of gallium-68 chloride produced via a cyclotron solid target system. The physicochemical characteristics of this product, with particular emphasis on pH and chemical purity, enable direct radiolabeling using commercially available kits and standard procedures developed for generator-based gallium-68. To our knowledge, this is the first report describing a gallium-68 chloride precursor obtained from the cyclotron solid target suitable for centralized production and distribution. Furthermore, this study provides an evaluation of [^68^Ga]Ga-PSMA-11 and [^68^Ga]Ga-DOTATOC preparations obtained using methods designed for generators, including commercially available kits originally optimized for generator-derived gallium-68. Based on the results presented herein, it was demonstrated that radiopharmaceuticals meeting the required quality specifications can be successfully produced. No statistically significant differences were observed in the imaging results obtained with the tested compounds, suggesting that the synthesis process and the formation of a stable radiometal complex effectively eliminate potential differences between the sources of gallium-68. Notably, the V-Ga68 precursor described in this work represents the first registered gallium-68 chloride radiopharmaceutical precursor produced using a solid target on a medical cyclotron.

The method for producing gallium-68 chloride described in this article was implemented in 2024 by Voxel S.A. for routine use in their V-Ga68 radiopharmaceutical precursor, intended for labeling kits for preparing radiopharmaceuticals in its nuclear medicine facilities for use in PET/CT imaging, which additionally confirms the full equivalence of the gallium-68 isotope obtained from the solid target and generator.

## 4. Materials and Methods

### 4.1. Reagents

All the reagents used in this study were of high quality grade. Zinc-68 metal powder (98.2% enriched) was purchased from Neonest (Solna, Sweden). Ultrapure water and 37% hydrochloric acid were purchased from Honeywell (Seelze, Germany). SPE separation cartridges ZR and TK200 were purchased from TRISKEM (Bruz, France); C18 cartridges were obtained from WATERS (Milford, MA, USA). For radiolabeling, the following precursors were used: precursor A: 10 µg PSMA-11 trifluoroacetate salt (ABX, Radberg, Germany), precursor B: 40 µg PSMA-11/Mannitol (piCHEM, Raaba-Grambach, Austria), precursor C: PSMA-11 kit for radiolabeling, 20 µg PSMA-11 (POLATOM, Otwock, Poland), precursor D: 50 µg DOTATOC acetate (ABX, Radberg, Germany), precursor E: 50 µg DOTATOC (acetate salt; piCHEM, Raaba-Grambach, Austria), precursor F: a kit preparation for 68Ga-labeling of DOTATOC, SomaKit TOC^®^ (Novartis, Rueil-Malmaison, France). Ascorbic acid and ethanol were purchased from Merck (Darmstadt, Germany). GalliaPharm ^68^Ge/^68^Ga Generator was obtained from Eckert Ziegler (Berlin, Germany). Media and supplements for cell culturing were purchased from Sigma-Aldrich (St. Louis, MO, USA), unless stated otherwise.

### 4.2. Cyclotron Production of Gallium-68 Chloride Solution for Radiolabeling

The cyclotron-produced gallium-68 chloride solution for radiolabeling was prepared at VOXEL Radiopharmaceutical Production Center in Kraków, and 50–70 mg of enriched zinc-68 (NEONEST) was electroplated on the platinum-made solid target shuttle. Subsequently, the shuttle was sent for irradiation to the PETtrace 860 cyclotron (GE Healthcare, Uppsala, Sweden) equipped with the ALCEO solid target processing system (COMECER, Castel Bolognese, Italy). The target was next irradiated with 12.0 MeV proton energy at 45 µA beam current for 60–180 min. After irradiation, the target deposit was dissolved and purified in the Taddeo synthesizer (COMECER) using ZR resin and TK200 cartridges. The resulting gallium-68 chloride solution was formulated with ultrapure 0.1 M hydrochloric acid, dispensed to vials, and terminally sterilized in a Theodorico 2 dispenser (COMECER). Quality control parameters of all batches used in the study were assessed using the following methods: radionuclide identity and purity were determined with a gamma spectrometer equipped with an HPGe detector (Canberra Meriden, CT, USA); radiochemical purity was analyzed using a TLC Mini-Scan system (BIOSCAN, Poway, CA, USA); iron and zinc content were measured by an in-house colorimetric test with a UV-VIS spectrophotometer (Shimadzu, Kyoto, Japan); and half-life was determined using a dose calibrator (COMECER). The final product was fully compliant with monograph 3109 of the European Pharmacopoeia for accelerator-produced gallium-68 chloride [17]. Compliance with all quality parameters was maintained over the entire 5-h shelf-life.

### 4.3. Labeling Procedure for [^68^Ga]Ga-PSMA-11 and [^68^Ga]Ga-DOTATOC

#### 4.3.1. [^68^Ga]Ga-PSMA-11 (Precursors A and B)

PSMA-11 precursor and 5 mg of ascorbic acid dissolved in 1 mL of acetate buffer were added to a vial containing about 5 mL of gallium-68 chloride in 0.1 M hydrochloric acid. The vial was placed in a heating block preheated to 95 °C and incubated for 5 min.

#### 4.3.2. [^68^Ga]Ga-PSMA-11 (Precursor C)

The labeling procedure of the precursor C was performed according to the standard kit manufacturer’s instruction. Five mL of gallium-68 chloride in 0.1 M hydrochloric acid was added to the kit vial. The vial was placed in a heating block preheated to 95 °C and heated for 10 min.

#### 4.3.3. [^68^Ga]Ga-DOTATOC (Precursors D and E)

DOTA-TOC precursor in 1 mL acetate buffer was added to a vial containing about 5 mL of gallium-68 chloride. The reaction mixture was placed in a heating block preheated to 95 °C and heated for 10 min. After cooling, the reaction mixture was applied to a C18 column, rinsed with 10 mL of water, and eluted with 1 mL of 50% ethanol in water to a vial containing 25 mg of ascorbic acid in 5 mL of phosphate saline buffer.

#### 4.3.4. [^68^Ga]Ga-DOTATOC (Precursors F)

The labeling procedure of the precursor F was performed according to the standard kit manufacturer’s instruction. Five mL of gallium-68 chloride in 0.1 M hydrochloric acid was added to the kit vial. Subsequently, 0.5 mL of the kit’s reaction buffer was added and the vial was placed in the heating block preheated to 95 °C for 7 min.

### 4.4. Labeling Procedure for [^68^Ga]Ga-PSMA-11 and [^68^Ga]Ga-DOTATOC for In Vivo Study

For the animal model study, the labeling procedure of [^68^Ga]Ga-PSMA-11 and [^68^Ga]Ga-DOTATOC preparations was performed at the University of Warsaw, Biological and Chemical Research Centre either with the generator eluate obtained on site, or with a gallium-68 chloride solution produced at the Radiopharmaceutical Production Center in Kraków and transported to Warsaw over a distance about 300 km. For this study, only the precursors A and D were used for [^68^Ga]Ga-PSMA-11 and [^68^Ga]Ga-DOTATOC preparations, respectively. In order to obtain the appropriate volume of a solution for administration to animals, after elution from the C18 cartridge, the sample was evaporated in an N2 flow and then reconstituted in 0.5 mL of phosphate buffer. Purity of the obtained preparations was verified by the radioTLC method.

### 4.5. Quality Control

#### 4.5.1. High-Performance Liquid Chromatography

The radiochemical purity of [^68^Ga]Ga-PSMA-11 and [^68^Ga]Ga-DOTATOC preparations was determined by the HPLC Shimadzu system equipped with Raytest Gabi Star radiometric and Shimadzu SPD-20A spectrophotometric detectors. The GL Sciences Intersil ODS-3 C18 (3 µm, 3.0 × 150 mm) column was used. The analyses were performed according to the relevant Ph. Eur. monographs [18,19].

#### 4.5.2. Thin Layer Chromatography

Thin layer chromatography (TLC) was performed as described in Eu. Pharm monograph 3044 and 2482 [18,19]. A 50:50 (*v*/*v*) solution of 0.77 g/L ammonium acetate and methanol was used as the mobile phase. The preparation was spotted directly onto the iTLC-SG paper and measured using a suitable detector. For radiochemical purity, TLC plate reader Miniscan TLC BIOSCAN scanner was used.

### 4.6. Cell Cultures

The human prostate cancer cell line LNCaP was cultured in RPMI-1640 with the addition of D-glucose (4.5 mg/mL), 10 mM HEPES, 1 mM sodium pyruvate, and 10% FBS. The rat pancreatic cancer cell line AR42J was cultured in F-12K medium (Kaighn’s modification of Ham’s F-12 medium; ATCC, Manassas, VA, USA) with the addition of 20% FBS. Both media were supplemented with penicillin (100 U/mL) and streptomycin (100 μg/mL). Cultures were maintained at 37 °C in a 95% humidified atmosphere with 5% CO_2_. After reaching 80–90% of confluence, the cells were washed with a PBS buffer and detached from the vessels with 0.25% trypsin-EDTA solution. The cells were collected, centrifuged at 250× *g* for 5 min at room temperature, and resuspended in a fresh medium without the addition of FBS and antibiotics.

### 4.7. Animal Models

Procedures requiring the use of laboratory animals were performed in accordance with the resolution of the 1st Local Ethical Committee for Animal Experiments in Warsaw (permission No. 1241/2021). In the experiment, 7-week-old male mice of the balb/c NUDE (BALB/cAnN-Foxn1*^nu/nu^*/Rj) obtained from Janvier Labs, 16 per each tumor type, were used. Mice were kept at a 12 h–12 h light cycle, humidity at the level of 55 ± 10%, and temperature of about 22 ± 2 °C in individually ventilated cages with unlimited access to drinking water and livestock mice diet. A mouse oncological model of expression of PSMA antigen was created by implantation of the animals with the suspension of 2 million cells of the LNCaP cell line in the basal membrane matrix. A mouse oncological model of SSTR receptor expression was created by implantation of the animals with the suspension of 2 million cells of the AR42J cell line in the basal membrane matrix.

### 4.8. PET/CT Measurements

Measurements of biodistribution of preparations were performed on a Bruker Albira small animal PET/SPEC/CT trimodal scanner. To compare the places of accumulation of [^68^Ga]Ga-PSMA-11 and [^68^Ga]Ga-DOTATOC preparations labeled with gallium-68 isotope of different origins, static PET measurements were performed. For this purpose, animals with a tumor of the appropriate kind and size were injected through the tail vein with doses of 16.7 ± 2.8 MBq and 15.4 ± 2.1 MBq of [^68^Ga]Ga-PSMA-11 and [^68^Ga]Ga-DOTATOC, respectively. Radioconjugates were synthetized with isotopes produced by cyclotron or generator origin. The measurement was started 60 ± 2 min after the injection. Imaging protocol includes 2 steps: PET acquisition and complementary CT imaging. PET was measured in stationary whole-body imaging mode with an acquisition time of 15 min. Whole body CT imaging was performed with the following parameters: 2 beds by 600 projections, tube voltage 45 kVp, current 400 mA. Animals were kept under isoflurane in oxygen anesthesia; breath rate and body temperature were monitored during imaging. The files generated during the measurement were reconstructed using the Albira Reconstructor software, version 5.8 (Bruker, Oncovision, Valencia, Spain) using the MLEM algorithm with 12 iteration and scatter, randoms, decay, normalization, and dead time corrections. PET and CT scans were fused using PMOD software, version 4.02 (PMOD Technologies LLC, Zurich, Switzerland). Tissue contour on fused image was prepared on all relevant slices. The obtained volumes of interest (VOI) were analyzed quantitatively, and the percent of injected dose per milliliter (%ID/mL) was calculated.

## 5. Conclusions

The study demonstrated that [^68^Ga]Ga-PSMA-11 and [^68^Ga]Ga-DOTATOC preparations obtained using gallium-68 chloride from both cyclotron solid target and generator sources present equivalent radiochemical purity. Furthermore, the analysis of gallium-68-labeled compounds’ accumulation in neoplastic lesions revealed no significant differences based on the radionuclide source for either [^68^Ga]Ga-PSMA-11 or [^68^Ga]Ga-DOTATOC. These findings provide strong evidence for the functional equivalence of gallium-68 isotope obtained from generator eluate and solid target cyclotron production. Moreover, the study confirms that kits validated for radiolabeling with generator-derived gallium-68 isotope can be equally labeled with cyclotron-produced gallium-68 isotope complying to specifications outlined in Ph. Eur. Monograph 3109 for use in PET/CT. This enables the direct use of the isotope obtained with the described technology in combination with commercially available kits for radiolabeling, facilitating the application of existing optimized procedures without the need for additional modifications.

## Figures and Tables

**Table 1:** Quality control results for [^68^Ga]Ga-PSMA-11 obtained using the cyclotron solid target gallium-68 chloride.

		Precursor A	Precursor B	Precursor C
**Peptide mass [µg]**		10	40	20
Appearance		Clear colorless	Clear colorless	Clear colorless
pH ^1^		4.05 ± 0.04	4.08 ± 0.04	4.37 ± 0.03
Radiochemical purity ^1^ TLC method [%]		99.75 ± 0.04	99.55 ± 0.38	98.75 ± 0.39
Radiochemical purity ^1^ HPLC method [%]		99.77 ± 0.20	99.30 ± 0.81	98.33 ± 0.22
Activity [MBq]	Run 1	2520	2090	2200
	Run 2	2080	1660	2200
	Run 3	1090	3095	1850

^1^ Mean ± SD for the three independent labeling runs.

**Table 2:** Quality control results for [^68^Ga]Ga-DOTATOC obtained using the cyclotron solid target gallium-68 chloride.

		Precursor D	Precursor E	Precursor F
**Peptide mass [µg]**		50	50	40
Appearance		Clear colorless	Clear colorless	Clear colorless
pH ^1^		4.12 ± 0.05	4.18 ± 0.01	3.28 ± 0.02
Radiochemical purity ^1^ TLC method [%]		98.18 ± 0.83	98.14 ± 1.22	98.22 ± 0.15
Radiochemical purity ^1^ HPLC method [%]		99.51 ± 0.61	99.53 ± 0.62	99.39 ± 0.07
Run 1	Activity [MBq]	1140	834	1210
	Yield [%]	61.29	53.81	N.A.
Run 2	Activity [MBq]	440	232	715
	Yield [%]	58.67	54.59	N.A.
Run 3	Activity [MBq]	475	392	490
	Yield [%]	62.50	54.07	N.A.
Yield ^1^ [%]		60.81 ± 1.96	54.15 ± 0.40	N.A.

^1^ Mean ± SD for the three independent labeling runs.

## Data Availability

The data are available from the authors.

## Supplementary Materials

The following supporting information can be downloaded at https://www.mdpi.com/article/10.3390/molecules30173458/s1: Table S1: Quality control results of representative batch of gallium-68 chloride produced by VOXEL Radiopharmaceutical Production Center.
