# Supplementary material for: Evaluation of Cyclotron Solid Target Produced Gallium-68 Chloride for the Labeling of [68Ga]Ga-PSMA-11 and [68Ga]Ga-DOTATOC
Source: Molecules. 2025 Aug 22;30(17):3458. doi: 10.3390/molecules30173458 (PMC12430084; doi:10.3390/molecules30173458)
Supplement: Supplementary file 1 [file molecules-30-03458-s001.zip › molecules-3763498-supplementary.pdf]

Table S1. Quality control results of representative batch of gallium-68 chloride produced by VOXEL Radiopharmaceutical Production Center.

Parameter	Method	Result
Visual appearance	Visual	A clear colourless solution
Identification	Gamma Ray spectroscopy	The principal gamma peak at 0.511 MeV; additional: 1.077 MeV, 1.022 MeV, 1.883 MeV
Identification	Dose calibrator	Half-life 67.8 minutes
pH	pH-meter	1.01
Radionuclide Purity [1]	Gamma Ray spectroscopy	Peaks in the gamma spectrum corresponding to photons with an energy different from 0.511 MeV; 1.077 MeV; 1.022 MeV and 1.883 MeV represent not more than 0.1 per cent of the total radioactivity
Radionuclide Purity [1]	Gamma Ray spectroscopy	The total radioactivity due to gallium-66 and gallium-67 NMT 2%
Radiochemical purity [1]	TLC	[^{68}Ga]gallium (III) ion: 100%
Chemical purity [1]	UV-VIS Spectrophotometry	8 μg Fe / GBq<br>8 μg Zn / GBq
Bacterial Endotoxins	LAL test	< 5 EU/ml
Sterility	Ph. Eur. method	Sterile

[1] Parameter met until the end of the product's shelf life.
